# Post-Prandial Changes in Salivary Glucocorticoids: Effects of Dietary Cholesterol and Associations with Bile Acid Excretion

**DOI:** 10.3390/nu11020360

**Published:** 2019-02-09

**Authors:** Graham W. Anderson, Christopher J. Kenyon, Emad A.S. Al-Dujaili

**Affiliations:** 1 Department of Dietetics, Nutrition & Biological Sciences, Queen Margaret University, Musselburgh EH21 6UU, UK; graham.anderson1991@gmail.com; 2 BHF Centre for Cardiovascular Science, Queen’s Medical Research Institute, University of Edinburgh, Edinburgh EH16 4TJ, UK; chris.kenyon2@btinternet.com

**Keywords:** glucocorticoids, cortisol, cholesterol, diet, bile acids

## Abstract

Mechanisms to explain post-prandial increases in circulating glucocorticoids are not well understood and may involve increased adrenal secretion and/or altered steroid metabolism. We have compared salivary levels of cortisol and cortisone levels in healthy male and female volunteers fed either a low or cholesterol-rich midday meal. Urinary levels of steroids, bile acids and markers of lipid peroxidation were also measured. Males and females showed expected circadian changes in salivary steroids and postprandial peaks within 1h of feeding. After a high-cholesterol meal, postprandial cortisol increases were higher in males whereas post-prandial cortisone levels were higher in females. Urinary cortisol but not cortisone levels were higher on the day when males and females ate a high-cholesterol meal. Urinary bile acid excretion and anti-oxidant markers of lipid peroxidation, thiobarbituric acid reactive substances (TBARS), and total phenol content were not affected by dietary cholesterol but tended to be higher in males. Cross-tabulation of correlation coefficients indicated positive associations between urinary markers of peroxidation, bile acids, and cortisol:cortisone ratios. We conclude that dietary cholesterol (a substrate for steroidogenesis) does not have an acute effect on adrenal glucocorticoid synthesis and that gender but not a high-cholesterol meal may influence the interconversion of cortisol and cortisone. Longer term studies of the effects of dietary cholesterol are needed to analyze the associations between bile acids, steroid metabolism, and secretion and lipid peroxidation.

## 1. Introduction

In addition to diurnal variations in circulating glucocorticoid hormone levels (high in the morning, low at night), it is well known that levels increase following the ingestion of a meal [1]. Although, the underlying cause of this postprandial effect is not well understood and may or may not involve ACTH [2,3], the response is likely to be physiologically important in the regulation of glucocorticoid hormone activities [4], particularly in relation to the control of intermediary metabolism.

Bearing in mind the known effects of glucocorticoids on carbohydrate, lipid, and protein metabolism [5], several studies have investigated whether the type of macronutrient influences the postprandial response [6,7,8,9]. Cortisol responses in saliva or plasma to meals with high protein content or high carbohydrate content have been claimed in different studies to be greater or to be unaffected [7,10,11,12,13]. Arguably, variability in responsiveness may be due to effects of gender or age or adiposity of participants [14] but discrepancies may also be because test meals are enriched mixtures of macronutrients rather than single types of macronutrient. To address this, a recent study considered the effects of feeding specific diets with single macronutrients [8], and the relative contributions of adrenal and extra-adrenal sources of glucocorticoid hormones to the increase in circulating cortisol levels after a midday meal were investigated. Deuterated steroid tracers were used to determine adrenal secretory activity, the clearance of the biologically active hormone cortisol, and the regeneration of cortisol from the inactive metabolite cortisone by the enzyme 11β-hydroxysteroid dehydrogenase type 1. Clearance was not affected by the type of macronutrient but protein intake had a larger effect on adrenal secretion than either carbohydrate or lipids, whereas carbohydrates had the largest effect on cortisol regeneration. 

Although these results are unequivocal, it is notable that cholesterol, a component of many food substances, was not a defined variable in any of the experimental protocols used to assess postprandial cortisol. Steroid hormones, which are not stored by steroidogenic tissues, are synthesized on demand using cholesterol as substrate. Cholesterol oxidation products are also potential competitive inhibitors of 11β-HSD1 activity. Bile acids which also derive from further metabolism of cholesterol are released into the intestine in response to food intake. One of the main functions of bile acids is to aid digestion but they are also recycled to the gall bladder via the circulation and, therefore, have the potential to influence cortisol levels by modulating steroid metabolism [15] and 11β-HSD1 activity [16] and by direct effects on adrenal steroidogenic gene expression via FXR (a transcription factor which is controlled by bile acids). However, although marked postprandial increases in circulating cholesterol have been demonstrated [17], particularly in response to a high fat meal, only a small fraction of this increase is derived acutely from dietary sources [18]. Longer-term studies indicate a reciprocal relationship between cholesterol uptake and synthesis [19]. A further factor to consider is the level of oxidative stress caused by feeding. Both steroidogenesis and steroid metabolism are susceptible to modulation by redox potential and lipid peroxidation [20,21]. Since it is known that markers of oxidative stress are increased postprandially [22,23], it follows that cortisol levels may, in turn be affected. 

Salivary steroid measurements are a convenient, non-invasive way of analyzing longitudinal physiological effects in volunteers and have been used by us to demonstrate pharmacological effects on steroid biosynthesis and metabolism [24,25]. However, bile acids and markers of lipid peroxidation are not measurable in saliva. Urinary measurements are a possible alternative although these measurements are better suited to analyzing the cumulative effects of daily treatments rather than the dynamic minute/hourly response to the ingestion of a meal. Accordingly, we have used saliva to identify acute postprandial changes in glucocorticoids and 24 h urine samples to look for changes in bile acids and for evidence of lipid peroxidation in relation to glucocorticoid excretion. Both cortisol and cortisone have been measured to assess their interconversion by the enzymes 11βHSD types 1 and 2. To test the effects of cholesterol, isocaloric meals have been designed with varied cholesterol content but broadly similar carbohydrate, protein and lipid composition. Both males and females have been tested because postprandial cholesterolaemia has been shown to be sex-specific [26].

## 2. Materials and Methods

### 2.1. Study Population/Recruitment

Volunteers (eight Males and eight Females) were recruited via face-to-face contact and recruitment emails. Each participant provided written, informed consent and the study was granted ethical approval from the Queen Margaret University (QMU) Board of Ethics. Project code: SFE studentship14/15-1100870. Date of approval, 6/6/2015 by QMU Central Research Ethics Committee. Ethics Approval Code HUBS3-1100870/DNBS/QMU. The study conformed to the guidelines set by the Declaration of Helsinki, and all participants provided written informed consent. Anthropometric details are summarised in Table 1.

### 2.2. Study Design

**Figure 1:**
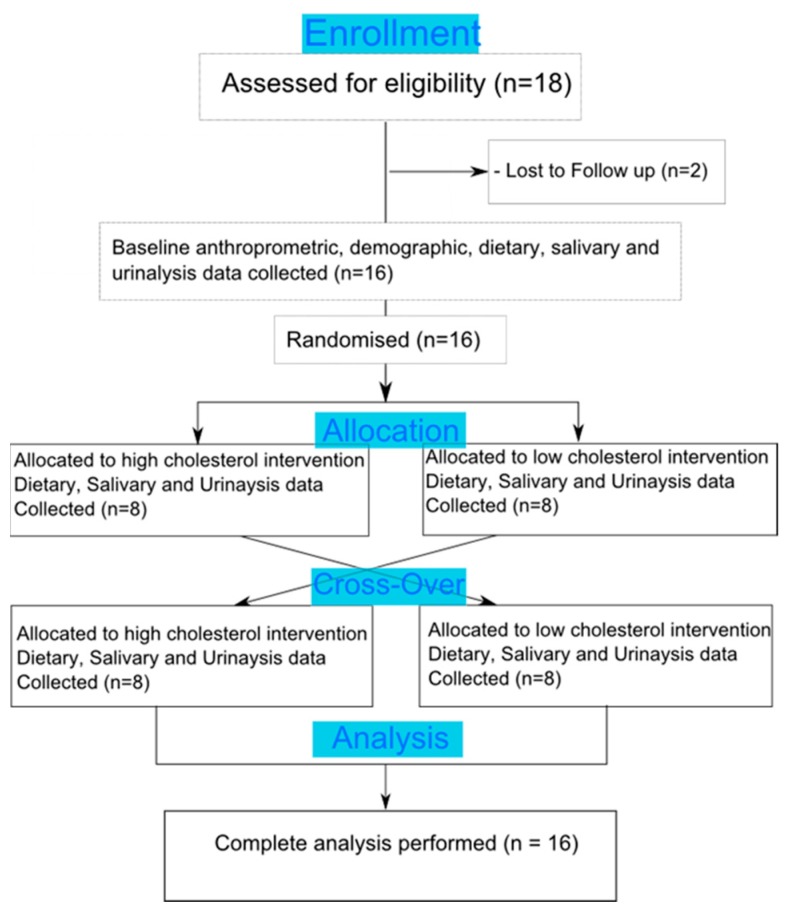
A flow diagram showing the cross-over design of the study.

The study followed a randomised cross-over design which involved the baseline measurements of dietary, physiological and metabolic markers followed by two interventions, separated by a minimum of three days wherein metabolic markers were revaluated. The randomisation procedure was performed by a technical staff independent of the study who allocated treatment type using an internet random number generator site, and unblinding took place after the study was complete and the allocations were emailed to the researcher. Figure 1 shows the flow diagram of the study. Each participant was randomly allocated to receive a midday meal with either a high cholesterol content (710 mg of cholesterol, 49 g fat, 39 g protein, 13 g carbohydrate, equivalent to 648 kcal) or a low cholesterol content (90 mg of cholesterol, 39 g of fat, 31 g protein, 48 g carbohydrate, equivalent to 652 kcal). Saliva and urine samples were taken throughout the day of each intervention. To estimate nutrient composition under basal conditions, each participant provided a three-day food diary (two weekdays and one weekend day) which was then analysed using the WinDiets software package (Robert Gordon University, Scotland, UK 2005). 

### 2.3. Saliva and Urine Sampling

On each intervention day, the participants provided saliva samples at six different time points: 30 min after awakening, 30 min before lunch, 15 min after lunch, 30 min after lunch, 90 min after lunch, and 30 min before bed. Twenty four–hour urine samples were also collected at baseline, high-cholesterol, and low-cholesterol meal days. All samples were coded and stored at −20 °C.

### 2.4. Cortisol/Cortisone Extraction & Analysis

Steroids were extracted from saliva and urine samples by vortexing with five volumes of ether (Sigma Aldrich). After freezing at −80 °C the ether was then decanted into test tubes and dried under a constant stream of nitrogen at 40 °C. Steroids were analysed by the in-house sensitive and specific ELISAs as described previously [24,25,27].

### 2.5. Urine Analysis—FRAP, TBARS, Total Phenolics, and Bile Acids

Ferric Reducing Antioxidant Power(FRAP), Thiobarbituric Acid Reactive Substances (TBARS), total phenol, and bile acids content of urine samples were analysed according to standard protocols [9,28,29,30]

### 2.6. Statistical Analysis

Data were analysed with Minitab software using a general linear model of ANOVA for effects of gender, diet and sample time. Pearson correlation coefficients for urine data were calculated. *p* < 0.05 considered to be significant. 

## 3. Results

The subjects in this study were young, non-obese and normotensive (Table 1). On average, the proportion of fat, protein and carbohydrate in their normal ad libitum diet was estimated to be similar to the content of test meals with no significant difference between males and females. The average participant diet consisted of 2500 kcal with a protein, fat and carbohydrate caloric intake ratio of 1:2.7:3.6 and an average cholesterol intake of 270 mg per day. The daily caloric intake was approximately 3.7 times greater than the test meals. The average cholesterol content of ad libitum foods was equal to or slightly more than the corresponding content of the low cholesterol meal. 

### 3.1. Postprandial Effects on Salivary Glucocorticoids

**Figure 2:**
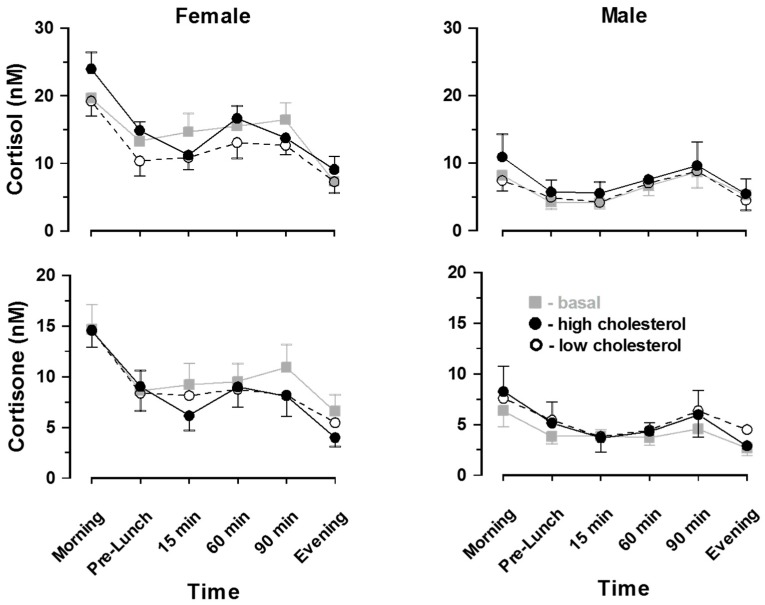
Salivary cortisol and cortisone values (mean ± SEM) showed expected diurnal variation with more marked increases in cortisol than cortisone. Female compared to male values were similar for cortisol, lower for cortisone and higher for cortisol:cortisone ratios (*p* < 0.01, see Table 2).

Salivary cortisol and cortisone values (Figure 2) show expected diurnal variations (morning versus evening) with more marked rhythms for females than males. Female compared to male values were higher for cortisol and cortisone (*p* < 0.001). Cortisol:cortisone ratios were higher in female than males (*p* < 0.01). On all experimental days, postprandial increases in cortisol and cortisone were noted for males and females. Overall, cortisol values were higher on the high compared with the low-cholesterol day (*p* < 0.01). However, postprandial cortisol responses to a high-cholesterol meal were not significantly different from those after a low-cholesterol meal in either female or male subjects (*p* > 0.1). Cortisone responses were not affected by diet. Despite non-significant effects of diet on either cortisol or cortisone the ratio of cortisol:cortisone (Table 2) was significantly greater after a high compared with a low-cholesterol meal (combined male and female post-prandial average ratio high-cholesterol diet, 1.95 ± 0.24 versus low-cholesterol diet, 1.56 ± 0.24; *p* < 0.01).

### 3.2. Urinary Steroids and Markers of Lipid Peroxidation

Irrespective of meals, urinary bile acids, total phenol, FRAP and cortisol levels are higher in males than females (*p* < 0.05) but TBARS and cortisone were not statistically different (Table 3). Two-way ANOVA indicated that compared with basal conditions, feeding a low- or high-cholesterol meal had no effect on urinary cortisol, bile acids, FRAP or total phenol but urinary TBARS, cortisone and cortisol: cortisone ratios were affected. In comparisons between high- and low-cholesterol days, TBARS values were 30% lower after a high compared with a low-cholesterol meal (*p* < 0.01), and cortisol:cortisone ratios were 25% greater (*p* < 0.05).

**Figure 3:**
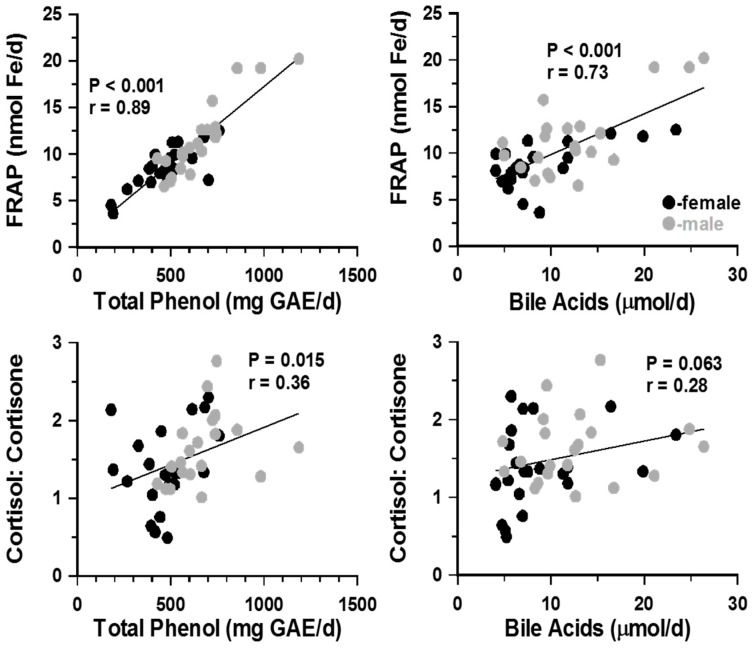
Cross-tabulation of urinary data showed that, irrespective of dietary intervention and gender, all three markers of oxidative stress were significantly correlated (*p* < 0.001 in all casesAlthough neither urinary cortisol nor cortisone alone were associated with antioxidant levels (not shown above), the ratio of cortisol: cortisone correlated positively with TBARS, total phenols (r = 0.47, *p* < 0.01) and FRAP (r = 0.32, *p* < 0.05). Urinary levels of bile acids also correlated positively with all anti-oxidant markers (r = 0.408, *p* ≤ 0.001 for TBARS; r = 0.702, *p* < 0.001 for FRAP and r = 0.659, *p* ≤ 0.001 for total phenols) and also with urinary cortisol:cortisone ratio (r = 0.46; *p* < 0.001). Although separately, correlation patterns in males and females showed similar trends, statistically significant correlations were seen only when male and female results were pooled. FRAP = Ferric reducing antioxidant power; TBARS= Thiobarbituric acid reactive substances; GAE = Gallic acid equivalent.

To look for possible associations between urinary parameters, correlation coefficients were cross-tabulated. Total phenol, FRAP and bile acid excretions were closely associated with each other (r = 0.78 and r = 0.73, *p* < 0.001; Figure 2). These associations were irrespective of gender. Changes in TBARS excretion did not correlate with any other urinary parameter (*p* > 0.3). Cortisol and cortisone values were not associated with either bile acids or lipid peroxidation markers. However, the urinary ratio of cortisol: cortisone showed positive association with total phenols (Figure 3) and weak, non-significant correlations (r = 0.28, *p* ≥ 0.06) with bile acid and FRAP excretion reflecting, perhaps, altered 11β-HSD1 and/or HSD2 activities rather than adrenal glucocorticoids secretion.

## 4. Discussion

We have tested whether the cholesterol component of a meal influences postprandial glucocorticoid responses. Salivary rather than plasma steroid measurements were made to allow sampling away from the laboratory and to avoid stressful venepunctures. Both cortisol and cortisone were assayed to assess whether variations in 11 β-hydroxysteroid dehydrogenase type 1 (11β-HSD1) activity modulated the outcome [8,31]. The initial hypothesis of this study was that cholesterol, as a substrate, might promote steroidogenesis. Bile acids, which are released from the gall bladder in response to food intake, are also derived from cholesterol. The synthesis of bile acids and the metabolism of steroid hormones are conflicted because both pathways involve some of the same enzymes [15]. Also, bile acids are recognized inhibitors of 11β HSD1 which regenerates the biologically active glucocorticoid, cortisol, from the inactive metabolite, cortisone [16,32]. Male and female subjects were assessed separately because several groups have noted gender differences in cortisol secretion and metabolism [16,33,34,35,36].

Salivary measurements confirmed that high- and low-cholesterol meals caused transient increases in cortisol which peaked at 15–60 min after eating. These increases were against the prevailing circadian decline. There was no consistent effect of dietary cholesterol. Based on previous reports of the distribution of cholesterol this might have been anticipated. Although the cholesterol content of the food was fivefold greater, it is unlikely that this would be available for steroidogenesis over the time course of salivary cortisol changes. Previous studies have shown that although a lipid-rich meal causes a prompt increase in plasma triglycerides and cholesterol levels, the increase in cholesterol is not due to immediate uptake from the diet [17,18]. Deuterium labelled cholesterol shows that uptake from food follows on after the peak in plasma cholesterol and comes after the peak in salivary cortisol which we observed in the present experiment. Interestingly, although total plasma cholesterol has been shown to increase in relation to the cholesterol content of meals, the cholesterol ester associated with lipoproteins declines [17]. Cholesterol for steroidogenesis comes from several sources including uptake from circulating lipoproteins [37]. It is possible, therefore, that the postprandial decline in HDL and LDL cholesterol esters that others have described could represent that portion taken up by the adrenal gland for steroidogenesis. It is notable that the meal-induced decline in lipoprotein cholesterol ester has been shown to be later than the expected postprandial increase in circulating glucocorticoid hormones.

The pattern of salivary cortisone secretion was broadly similar to that of cortisol: evidence of a postprandial increase but no effect of treatment. Overall, female cortisone levels tended to be greater than male values, but gender specific responses to low and high cholesterol meals were not affected. Averages of individual cortisol:cortisone ratios at each time point were similar in females and males (i.e. no evidence of a gender effect on 11β-HSD1 and/or 11β-HSD2 activities). In a previous study, Stimson et al used labeled steroid to track adrenal secretion rates and to indicate in vivo rates of postprandial cortisol and cortisone inter-conversion. They demonstrated that the postprandial plasma cortisol increase was mostly from de novo adrenal biosynthesis with contributions from the regeneration of cortisol [31]. This fact has been widely accepted in relation to the components origin of body cholesterol being mostly from de novo synthesis. In the present study the postprandial ratios of cortisol:cortisone were significantly greater after a high-cholesterol meal than a low=cholesterol meal although the difference was significant in males (*p* < 0.001) but not in females (*p* > 0.1).

Differences in male and female salivary glucocorticoid levels were not consistent with those found in urine samples and with many other studies showing increased HPA activity in males. It should be noted, however, that the effects of stress and altered adrenocortical activity is seen in urine after a lag of several hours from the time of responses in adrenal, plasma and saliva. Bearing in mind also that the uptake of cholesterol from a meal extends beyond the initial postprandial rise in glucocorticoids [18], we assayed steroids in urine samples that included an 18 h postprandial period. Interestingly urinary cortisol levels tended to be reduced after a high-cholesterol meal. Clearly this response is not consistent with the idea that dietary cholesterol provides substrate for steroidogenesis. An alternative explanation might be that dietary cholesterol increased lipid peroxidation, which is known to have an inhibitory effect on steroidogenic enzyme activity [20]. The effects of a high fat meal on markers of lipid peroxidation and commensurate changes in antioxidant levels are well established. Similarly, we have shown previously that increases in urinary antioxidants are induced by dietary polyphenols [25]. However, in the present experiment, although FRAP and total phenols correlate closely with one another, there was no effect of diet and there was no association with urinary cortisol or cortisone levels. Also, although not correlated with FRAP or total phenols values, the excretion of TBARS was decreased not increased after feeding a high-cholesterol meal. Arguably this could reflect the delayed decrease in circulating cholesterol esters that have been described following a high-cholesterol meal [18]. There were sex differences in urinary cortisol and both FRAP and total phenols but not TBARS. This probably explain why urinary cortisol:cortisone ratios correlated with urinary total phenols excretion but not TBARS.

The postprandial release of bile acids from the gall bladder is controlled by cholecystokinin and appears to be modulated by the volume and lipid content of food ingested [6]. Most bile acids are recycled from the gut and only a small fraction is excreted in urine and feces. Increases in serum bile acids peak at 2 h after oral intake of carbohydrate or lipids before hepatic re-uptake. This increase in serum bile acids would appear to be too late to influence the postprandial rise in salivary cortisol. However, it is notable that in the present experiment, urinary bile acids correlated with urinary FRAP and total phenol antioxidant levels and showed a trend for correlation with urinary cortisol:cortisone ratios. Again, gender rather than the cholesterol content of food appeared to be the main determinant of variation. The higher values in male than in female urine output contrast with previous findings in serum where concentrations of conjugated bile acids were generally higher in females than males [38] although higher female values were only apparent in those taking oral contraception. Although half of the females were not taking oral contraception in the present study, this still does not account for lower female urinary values. It is possible that male–female differences in urinary bile acid and steroid excretion and total phenol levels might all be related to gender differences in renal function.

## 5. Conclusions

A high-cholesterol meal did not affect postprandial levels of salivary cortisol. This may be because the cholesterol content of food was not immediately available for steroidogenesis. Urinary cortisol values collected over the day of feeding a high-cholesterol meal were not significantly affected compared to values on a low-cholesterol day. However, urinary TBARS, a marker of lipid peroxidation, was decreased. The TBARS response is consistent with known effects of dietary cholesterol intake on cholesterol ester levels. Reduced peroxidation and cholesterol ester levels would have confounding effects on steroidogenesis. Marked gender-specific differences in urinary levels of bile acids, FRAP and total phenol markers and cortisol:cortisone ratios are probably the reason why some of these parameters correlate with each other. Associations between these parameters might relate to sex-dependent differences in renal function. Future studies should look at longer duration of intervention and a larger number of subjects.

## Figures and Tables

**Table 1:** Anthropometrical and physiological characteristics and average daily macronutrient intake of male and female volunteers. Values shown are means ± SD.

Gender (*n*)	Female (8) Mean (SD)	Male (8) Mean (SD)
Age (years)	22 ± 0.6	23 ± 0.5
Weight (kg)	62.9 ± 3.8	70.7 ± 4.7
Height (m)	1.66 ± 0.02	1.75 ± 0.01
BMI (kg/m^2^)	22.8 ± 1.5	22.7± 1.3
Systolic BP (mmHg)	121 ± 1.9	125 ± 3.5
Diastolic BP (mmHg)	70 ± 2.7	71 ± 3.5
Basal Caloric intake (kcal/day)	2432 ± 201	2649 ± 394
% Carbohydrate intake	64.1 ± 2.5	65.5 ± 2.8
% Fat intake	17.7 ± 1.5	18.4 ± 1.9
% Protein intake	18.2 ± 1.4	16.1 ± 1.5
Basal Cholesterol (mg/day)	261 ± 48	269 ± 42

**Table 2:** Effects of sex and meals on salivary cortisol: cortisone ratio values in male and females.

**Female Cortisol:Cortisone**							
**Collection Time**		**AM**	**PRE**	**15 min**	**30 min**	**90 min**	**PM**
Basal	Mean	1.57	1.99	1.90	1.97	1.70	1.48
	± SD	0.30	0.31	0.32	0.31	0.23	0.29
High Cholesterol	Mean	1.68	1.91	2.16	2.87	1.90	2.63
	± SD	0.17	0.26	0.37	0.81	0.27	0.33
Low Cholesterol	Mean	1.43	1.56	2.26	1.66	1.69	1.42
	± SD	0.14	0.30	0.88	0.39	0.19	0.22
**Male Cortisol:Cortisone**							
**Collection Time**		**AM**	**PRE**	**15 min**	**30 min**	**90 min**	**PM**
Basal	Mean	1.39	1.09	0.99	1.69	1.86	1.96
	± SD	0.31	0.21	0.15	0.17	0.33	0.54
High Cholesterol	Mean	1.28	1.12	1.53	1.47	1.86	1.76
	± SD	0.14	0.14	0.10	0.17	0.22	0.16
Low Cholesterol	Mean	0.98	1.00	0.93	1.37	1.31	1.00
	± SD	0.14	0.14	0.10	0.17	0.22	0.16

Meal intake did not significantly affect values in either males or females. Overall, irrespective of diet, ratios were higher for females than males (*p* < 0.01). AM = morning (before breakfast); Pre = pre-lunch; PM = evening (before bedtime).

**Table 3:** Analysis of bile acids, glucocorticoids and oxidative stress markers in 24 h urine samples collected on basal, low-cholesterol, and high-cholesterol days (mean ± SD).

	Female			Male		
	Basal	Low Cholesterol	High Cholesterol	Basal	Low Cholesterol	High Cholesterol
Bile Acids (µmol/day)	8.22 ± 1.37	8.22 ± 1.87	9.64 ± 2.47	13.91 ± 2.18	11.88 ± 2.61	12.46 ± 1.49
Total Phenol (mg GAE/day)	469.8 ± 61.9	478.2 ± 41.9	479.8 ± 67.2	697.7 ± 67.4	655.5 ± 97.2	634.8 ± 36.5
FRAP (mmole Fe^2^/day)	8.36 ± 0.85	8.83 ± 0.74	8.68 ± 1.01	13.11 ± 1.91	11.33 ± 1.71	10.35 ± 0.57
TBARS (µmol/day)	3.65 ± 0.47	2.98 ± 0.5	1.96 ± 0.17	3.22 ± 0.27	3.43 ± 0.34	2.48 ± 0.21
Cortisol (nmol/day)	57.7 ± 6.56	60.4 ± 8.91	68.6 ± 12.0	78.9 ± 11.6	89.0 ± 12.2	69.1 ± 10.5
Cortisone (nmol/day)	32.2 ± 2.9	61.4 ± 7.34	52.7 ± 6.21	45 ± 6.34	64.7 ± 12.0	45.3 ± 8.51
Cortisol:Cortisone	1.79 ± 0.14	1.02 ± 0.12	1.34 ± 0.19	1.76 ± 0.15	1.45 ± 0.12	1.65 ± 0.22

FRAP = Ferric reducing antioxidant power; TBARS = Thiobarbituric acid reactive substances; GAE = Gallic acid equivalent.

## Abbreviations

ACTH	Adrenocorticotropic hormone
11β-HSD1	11β-Hydroxysteroid dehydrogenase type 1
ELISA	Enzyme linked immunosorbant assy
FRAP	Ferric reducing antioxidant power
GAE	Gallic acid equivalent
GC	Glucocorticoids
TBARS	Thiobarbituric acid reactive substances
